# A Double-Deck Self-Digitization Microfluidic Chip for Digital PCR

**DOI:** 10.3390/mi11121025

**Published:** 2020-11-24

**Authors:** Gangwei Xu, Huaqing Si, Fengxiang Jing, Peng Sun, Dan Zhao, Dongping Wu

**Affiliations:** 1 State Key Laboratory of ASIC and System, School of Microelectronics, Fudan University, Shanghai 200433, China; 18112020050@fudan.edu.cn (G.X.); 17112020035@fudan.edu.cn (H.S.); 18112020068@fudan.edu.cn (P.S.); zhaodan@fudan.edu.cn (D.Z.); 2 Turtle Technology Company Limited, Shanghai 200439, China; fengxiangjing@turtle-tech.cn

**Keywords:** double-deck, microfluidic chip, digital polymerase chain reaction

## Abstract

In this work, a double-deck microfluidic chip was presented for digital PCR application. This chip consists of two reverse-placed micro-patterned polydimethylsiloxane (PDMS) layers between the top and bottom glass substrates. Each micropatterned PDMS layer contains more than 20,000 cylindrical micro-chambers to hold the partitioned droplets. The double-deck designs can double the number of chambers and reagent capacity without changing the planar area of the chip. In addition, carbon black was mixed into the pure PDMS gel to obstruct the passage of fluorescence from the positive chambers between the two layers of the chip. Thus, the fluorescence signal of micro-chambers in different layers of the chip after PCR can be collected without mutual interference. The quantitative capability of the proposed chip was evaluated by measuring a 10-fold serial dilution of the DNA template. A high accuracy of the absolute quantification for nucleic acid with a dynamic range of 10^5^ was demonstrated by this chip in this work. Owing to its characteristics of small planar area, large capacity, and sensitivity, the double-deck microfluidic chip is expected to further promote the extensive applications of digital PCR.

## 1. Introduction

Digital PCR (dPCR) is an absolute quantitative method for nucleic acid with high precision. The strategy for dPCR is based on dividing samples into thousands or even millions of separate partitions, and the absolute concentration of the target molecule is then directly acquired according to the proportion of positive or negative partitions [1]. To subdivide the samples into a large number of partitions, various methods have been proposed to generate monodisperse micro-droplets in droplet microfluidic platforms (ddPCR platforms) and physical partitions microfluidic platforms (pdPCR platforms) in past decades [2,3,4,5,6].

Existing technologies have been seminal advances in sample compartmentalization. Among them, ddPCR platforms that have achieved the largest numbers of total partitions are particularly popular [7,8,9,10]. However, most of them require a complicated workflow and a droplet readout system. Worse yet, the real-time detection and addressability of individual droplets in ddPCR platforms are still challenging [11]. By contrast, pdPCR platforms achieve partitions by compartmentalizing samples into a large number of small immobilized chambers [2,12,13]. Compared with ddPCR platforms, pdPCR platforms have some unique advantages. For example, highly uniform and stable micro-aliquots can be generated in immobilized chambers of pdPCR platforms [14]. The immobilized chambers can avoid the coalescence of droplets caused by thermal oscillation [15]. Moreover, the partitions in pdPCR platforms can be concurrently detected and enable addressability [16]. What is more, pdPCR chips can be directly imaged through relatively simple fluorescence microscope systems, and the chip can be preserved for a long time after amplification, which enables a flexible experimental operation and data validation [15,16]. However, being limited to the area of a PCR instrument, chip area and throughput are inconsistent in terms of common sense. Research shows that the sensitivity of dPCR depends on the total volume of the reaction, which is determined by the number of partitions and their volume [17,18,19]. In some fields, especially at low viral load for the quantification of RNA, dPCR is less sensitive than qPCR, attributing to the lower total reaction volume [18,19,20]. As far as is known, existing pdPCR platforms all have a single-layer structure [3,13,15,16,21,22,23,24], and most of these chips have a lower total reaction volume [14,15,21,24], while these microfluidic chips can generate more droplets by increasing chip size, which will have to sacrifice throughput during dPCR amplification. Increasing the total reaction volume and number of partitions in the same chip area is a great challenge.

Inspired by multi-layer printed circuit boards (PCBs) and integrated circuit (IC) packages, a double-deck microfluidic chip is proposed in this work to increase the number of chambers and reagent capacity without changing the planar area of the chip. With the design of the double-deck structure, the microfluidic chip doubled the number of chambers and reagent capacity. To obtain fluorescence signals from the chambers in the chip, the top and bottom surfaces were transparent glass. In addition, carbon black was mixed into the pure PDMS gel to obstruct the passage of fluorescence from the positive chambers between the two layers of the chip. Thus, the fluorescence signal of micro-chambers in different layers of the chip after PCR could be collected without mutual interference. Finally, the quantitative performance of the proposed chip was verified with a serial dilution of the DNA template, and the result matched the expectation well.

## 2. Materials and Methods

### 2.1. Microfluidic Chip Design and Fabrication

**Figure 1:**
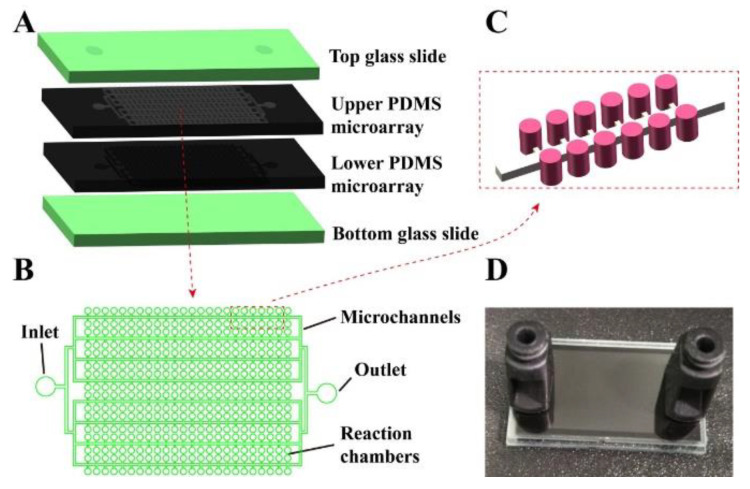
The double-deck microfluidic chip. (**A**) Schematic of the layered chip structure. (**B**) Schematic of microfluidic digital PCR (dPCR) chip. (**C**) Diagram of the details of the chip. (**D**) Photograph of the prototype device. The chip size is 35 mm × 20 mm.

The proposed self-digitization double-deck dPCR chip is depicted in Figure 1. The microfluidic chip consists of four layers: The top and bottom transparent glass slides as vapor barriers and signal-collecting windows, and two back-to-back micro-patterned PDMS layers with channels and chambers (shown in Figure 1A). The upper and lower chamber arrays are fabricated using the same mold and are connected through the inlet/outlet holes. As shown in Figure 1B, the microstructure of the chip is designed with a series of cylindrical sample cavities (chambers) evenly distributed along the two sides of the main microfluidic channel. The diameter of the cylindrical chambers is 87 μm and the height 120 μm. The height of the main channel is 50 μm and the width 60 μm. The width and length of the branch channel connected with the cylindrical chamber and main channel are 25 μm, and its height is 60 μm. Each layer of the chip contains 21,384 independent 0.71 nL micro-chambers, and the chip thus allows more than 30 µL of reagent to be delivered. The chip has a small footprint (35 mm × 20 mm), and the physical picture of the chip is shown in Figure 1D.

A two-level master was fabricated on a 4 in. (10.16 cm) silicon wafer covered with SU-8 (Microchem, MA, USA) by multi-layer soft lithography (see Supporting Information). The structural layer of the chip was made of PDMS (RTV-615; GE Advanced Materials). The simple process of chip fabrication is presented in Figure S1. First, premixed 10:1 (part A:part B) PDMS was mixed with carbon black at a 0.8% weight percentage. Subsequently, the mixture was de-gassed in vacuum, and a 4 mL mixture was then injected over the master using a syringe and cured at 100 °C for 10 min. After being cured, the PDMS was peeled off the master and holes were punched for an inlet/outlet. The thickness of the PDMS layer was 4 mm and the diameter of the inlet/outlet was 8 mm. Then, two PDMS layers were bonded to a clean top glass slide (with an inlet/outlet) and a bottom glass slide (without an inlet/outlet) by plasma treatment. Two PDMS-glass blocks were then bonded together, as shown in Figure S1D–F. The assembled chip was fully cured at 100 °C for 30 min, enhancing the bond strength between components of the chip. Finally, silicone valves were bonded to the inlet and outlet of the top glass slide reversibly to control the sample-loading process automatically.

### 2.2. Chip Operation

**Figure 2:**
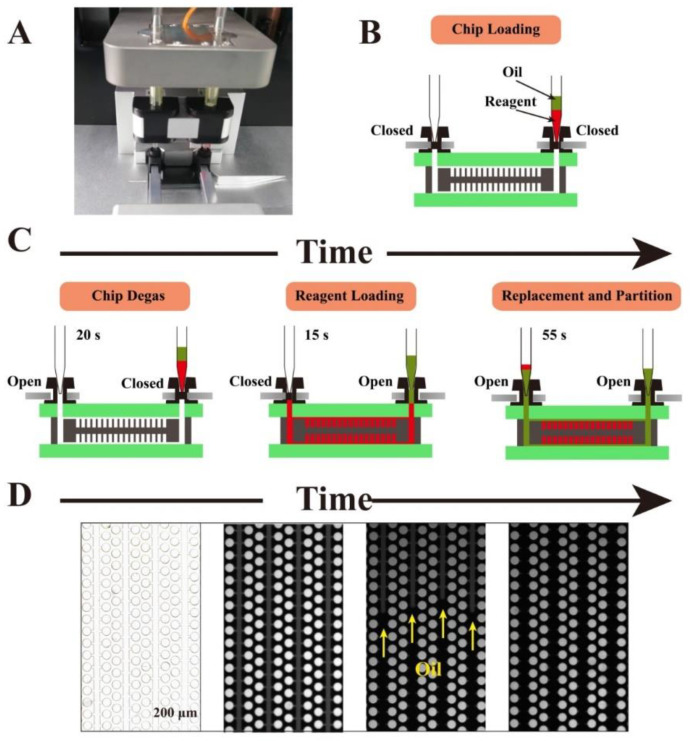
Cross-sectional view of chip operation process. (**A**) Photograph of the chip loader device. (**B**) Loading the chip in the chip loader device. (**C**) Degassing the chip in a vacuum pump attached to its outlets and then aspiration of the sample into the microchannels under the negative pressure and the oil into the microchannels to replace and partition the samples. (**D**) Micrographs of the microfluidic dPCR chip before sample loading (optical micrographs), after reagent loading, and after partitioning.

First, the oil phase was prepared at the ratio of 3A:1B in a 2 mL tube and mixed well by vortexing. Blended oil should be placed in ice to remain stable and uncured. After that, the reagent was prepared according to the ratio described below. To simplify the operation process, the dPCR chip was loaded by an automatic dPCR chip loader (Biodigital™, Turtle Tech Ltd., Shanghai, China). The basic operating procedure of the double-deck chip is outlined in Figure 2. The process of chip de-gassing, reagent loading, and sample partitioning was automatically in the chip loader. After chip loading was completed, the chip was taken out and the valves removed. Finally, the chip was transferred to a flat PCR apparatus (Turtle Tech Ltd., Shanghai, China) for digital PCR assay. One flat PCR apparatus can perform 10 chips in parallel.

### 2.3. PCR Amplification

For digital PCR amplification, all components of the PCR mixture, including the PCR master mix, primers, probe, and template, were pre-mixed off-chip before dPCR analysis. To assess the performance of the proposed dPCR chip, KRAS wild DNAs were serially diluted to yield dilutions ranging from 7 × 10^1^ to 7 × 10^5^ copies/μL. The dPCR reaction mixture contained a 10 × BioDigital dPCR Mix 6 (μL), KRAS forward primer (2 μL) (400 nM), KRAS reverse primer (2 μL) (400 nM), KRAS probe (2 μL) (400 nM), a serially diluted template (6 μL), and RNase-free water (42 μL). All DNA samples and reaction mix were stored at −20 °C prior to use. All primers and probers are listed in Table S1. The chip was then placed on a flat PCR apparatus for PCR reaction. The thermocycling protocol included a 10 min heating step at 50 °C to ensure that the oil was well solidified, and a 10 min “hot start” step at 95 °C to activate the Taq DNA polymerase, and then 45 thermal cycles were performed (20 s at 95 °C, 40 s at 58 °C) to amplify the target KRAS DNA. The experiments were repeated three times to ensure the robustness and reproducibility of the double-deck chip.

### 2.4. Image Acquisition and Analysis

All bright-field images were acquired with an inverted microscope (XDS-800C, Caikon, Shanghai, China), and all fluorescence images were acquired and analyzed using a monochrome Biochip reading system provided by Turtle Tech. Ltd. (Shanghai, China). The fluorescence was excited at 620 nm and the emitted light was accepted by the CCD through a 660 nm long-pass filter.

## 3. Results and Discussion

### 3.1. Effect of Double-Deck Design on Sensitivity and Detection Dynamic Range of dPCR Chip

For a dPCR, the sensitivity or lower limit of detection corresponds to the detection of a single molecule in a single partition. Hence, the minimal concentration that can be detected for a dPCR platform depends on the total volume of the reagent, which is determined by the number of partitions and their volume. Higher sensitivity requires larger total volume of the reagent. The dynamic range of detection is defined by the difference between the upper and lower limits of detection. The upper limit of detection is mainly determined by the number of partitions. The lower detection limit or the sensitivity is determined by the total volume [25].

Increasing the total volume of reaction is an effective way of acquiring high sensitivity and a wide dynamic range of detection. For traditional single-layer pdPCR platforms, larger total volume requires a larger chip footprint, which is a potential limitation for point-of-care purposes. At the same time, a larger chip footprint will reduce the throughput of dPCR detection. In this work, a double-deck structural microfluidic chip was proposed to increase the number of partitions and total reaction volume, and to improve the space utilization rate of the chip. Compared with a single-layer chip, the double-layer chip can double the number of chambers and reagent capacity at the same size, which is significant in improving the sensitivity and nucleic acid quantitative dynamic range of the dPCR. The upper and lower chamber arrays can allow thermal cycling at the same time, because the PDMS membranes between the two arrays of reaction chambers are very thin. As shown in Figure 1 and Figure S1, two layers of PDMS are placed back-to-back and the arrays of the two layers are bonded to the top and bottom glass slides. Therefore, the microfluidic chip has two arrays in different layers in three dimensions. As a result, the number of chambers of the dPCR is doubled, maintaining the same chip footprint.

In this work, a single layer of the chip contains 23,184 chambers, and the comparison between double-deck and single-layer chips in sensitivity and dynamic range of detection is shown in Table 1. The double-deck dPCR chip has a lower detection limit compared with the single-layer chip, because the number of chambers is doubled. The upper limit of detection is also increased as a benefit of the increase in chamber number. As a result, the dynamic range of the double-deck dPCR chip is more than twice that of the single-layer chip. In summary, the proposed double-deck dPCR chip increases the sensitivity and dynamic range of detection without increasing the chip footprint. The simple structure, small size, and large capacity make the chip easy to operate and suitable for aqueous sample digitization.

### 3.2. Sample Partition of dPCR Chip

The principle of the method used to partition the dPCR chip is shown in Figure 2. First, the chip is manually placed into the tray according to the instrument operation instructions. Second, a two-level micropipette is used to suck 50 μL of oil and 50 μL of reagent successively, and the tip with oil and reagent is then put in the inlet holder (shown in Figure 2B). Another empty tip is placed in the outlet holder to store the redundant reagents that will be replaced by oil. Then, the sample loading button is opened according to the operation manual, which is followed by the device automatically completing the follow-up processes (Figure 2C). First, the inlet valve is closed and the outlet valve is opened, and the pump then works to vacuum the chip at 0.5 KPa. Owing to the very small internal volume of the chip, the vacuuming process will be completed after 20 s. The outlet valve is then closed and the inlet valve is opened successively. Driven by the negative pressure inside the chip, the aqueous solution is sucked into the main channel and chambers quickly. After 30 s, the upper and lower chamber arrays will be filled with aqueous phase evenly (Figure 2C). Then, the outlet valve is opened and a positive pressure (131 KPa) is exerted immediately to push the continuous oil phase into the chip channel. Once the oil has been sucked into the channel, it will replace the aqueous phase in the channels of the chip (Figure 2C). Thus, all chambers filled with the sample in the chip will be separated by oil (Figure 2D). As the upper and lower arrays are connected through the inlet/outlet, they will be partitioned concurrently.

### 3.3. Uniformity Analysis of dPCR Chip

**Figure 3:**
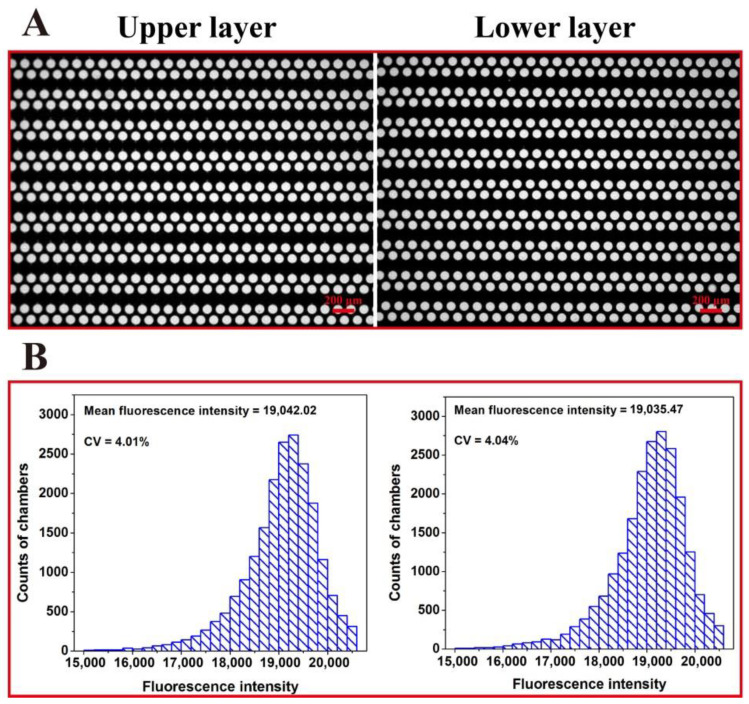
Fluorescent image of the upper and lower (**A**) arrays after loading fluorescence reagent, and uniformity analysis of the dPCR chip (**B**).

In a double-deck microfluidic chip, the upper and lower arrays are connected through the inlet/outlet and will be partitioned concurrently. The uniformity of the sample distributed in each chamber was investigated. A fluorescent dye was added into the chip, and the fluorescence photographs were then acquired by a BioDigital dPCR reader (Turtle Tech Ltd., Shanghai, China); the fluorescence intensity value of each chamber was measured by ImageJ. Figure 3A is a fluorescence microscope photograph of the chip after sample dispersing. Figure 3B shows the distribution of the fluorescence intensity values of the chambers in the upper layer and of the chambers in the lower layer. Statistical analysis of the distribution of fluorescence intensity values of the chambers in the upper and lower layers shows that the average fluorescence intensity of the chambers in the upper and lower layers is similar. In addition, the relative standard difference in the fluorescence-intensity value of the chambers in the upper and lower layers was approximately 4%, indicating that the micro-chambers were filled equally and the reagents partitioned uniformly.

### 3.4. Function of Black PDMS of Chip

**Figure 4:**
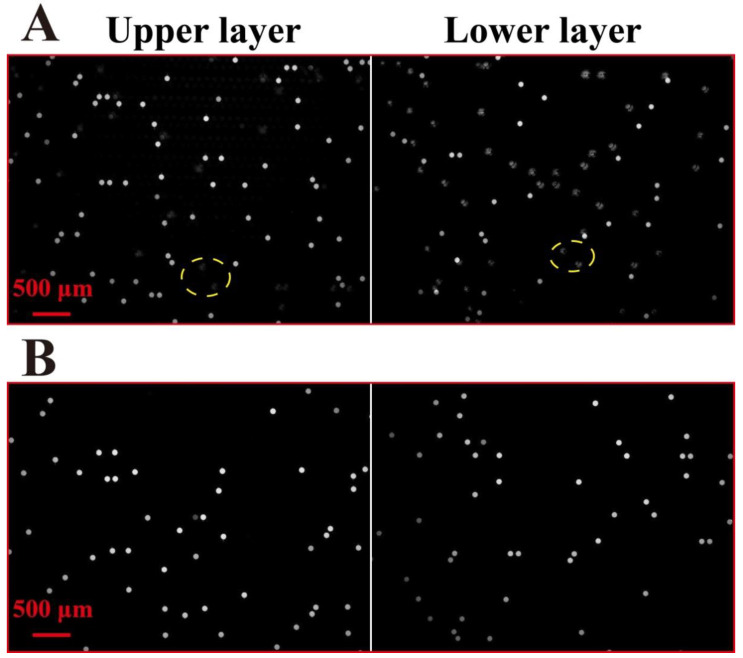
Fluorescence images of KRAS DNA molecule amplification on the transparent PDMS chip (**A**) and black PDMS chip (**B**).

In most traditional dPCR chips [11,24,26,27], the structural layer is transparent PDMS. A double-deck chip fabricated by transparent PDMS is not suitable for dPCR application, because of fluorescent signal interference between different layers. To eliminate the issue, black PDMS is used in the double-deck chip. To investigate the function of the black PDMS in the double-deck dPCR chip, comparison of fluorescence images between transparent and black PDMS double-deck chips after PCR amplification was performed. The KRAS wild DNA template with a concentration of 7 × 10^3^ copies/μL was used, and the CY5 fluorescence channel was selected. The comparison results of both upper and lower layers of the chambers of different PDMS chips are shown in Figure 4. There is mutual interference between the fluorescent signals from the different layers of the transparent PDMS double-deck chip, as shown in Figure 4A. The yellow circles indicate the transmitted fluorescence signal from the other layer. Unlike the transparent PDMS double-deck chip, no mutual interference was found in the black PDMS double-deck chip (shown in Figure 4B), clearly indicating that the double-deck chip using black PDMS is suitable for dPCR application.

### 3.5. Absolute DNA Quantification by dPCR

**Figure 5:**
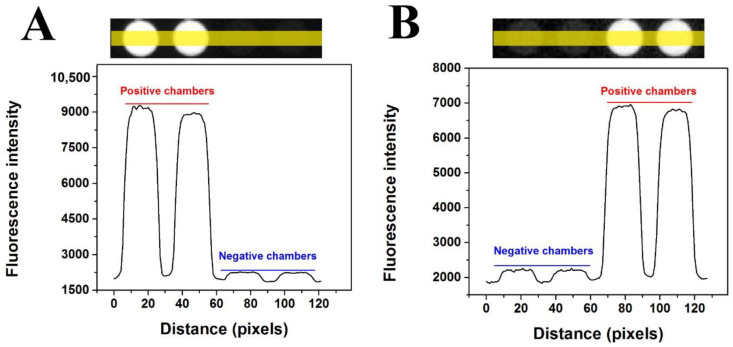
Fluorescence intensity contrast between positive and negative chambers of KRAS wild probes-CY5 in the upper (**A**) and lower (**B**) layers of the chip.

One of the most important applications of dPCR is the absolute quantification of nucleic acid independent of the standard amplified curve. Before conducting quantitative experiments, the feasibility of the double-deck dPCR chip was verified. Comparison of fluorescence intensity between positive and negative chambers after PCR amplification was performed. The fluorescence intensity contrast between positive and negative chambers is shown in Figure 5, in which the curve below the chambers shows the change in corresponding fluorescence intensity with the change in position, and the yellow line is the statistical area of fluorescence intensity. The fluorescence intensity of positive chambers is approximately sevenfold that of the negative chambers in the upper and lower layers of the proposed chip.

**Figure 6:**
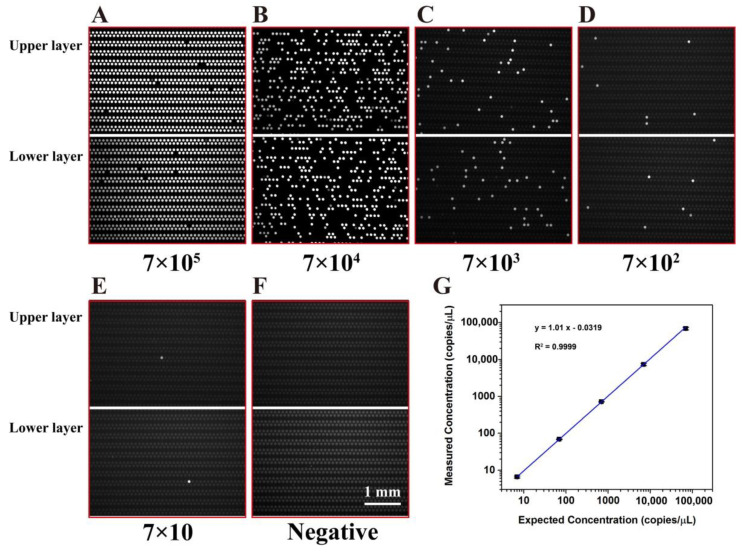
Digital PCR on the microfluidic dPCR chip with different concentrations of KRAS DNA: (**A**–**E**) Digital PCR on the dPCR chip with a serial dilution of target DNA template ranging from 7 × 10^5^ to 7 × 10 copies/μL. (**F**) The negative assay was in control when no target template was loaded. (**G**) The linear relationship between the measured concentration in the dPCR chip and the expected DNA concentration.

To evaluate the quantitative detection ability of the double-deck dPCR chip, KRAS wild gDNA templates were used here. The concentration of nucleic acid is calculated according to the number of positive chambers at the end point of the PCR. A serially diluted KRAS wild DNA template was used to perform a dPCR in the proposed chip, the concentrations of which ranged from 7 × 10^1^ to 7 × 10^5^ copies/μL to verify the quantitative ability of the chip. Several representative images of the dPCR chips after PCR amplification are shown in Figure 6A–D. As the DNA template in the PCR mixture solution was diluted serially, the fraction of positive chambers decreased proportionally. In addition, the negative controls did not have any positive chambers, evidencing that there was no contamination in all reactions (Figure 6E). According to the digital PCR concept, the concentration of the DNA in the PCR mixture solution can be calculated from the fraction of positive reactions using Poisson statistics, according to the following equation:

(1) $$\mathrm{Concentration}\;=\;-\frac{ln\left(1-\frac{d}{n}\right)}{V}$$

where *n* is the total number of effective chambers in each dPCR chip, *d* the number of positive chambers, *d/n* the fraction of positive chambers, and *V* the single volume of a chamber (μL).

The results show that the measured concentration of the DNA can match well the expected concentration of the DNA (R^2^ = 0.9996) (Figure 6F).

## 4. Conclusions

In this work, a double-deck array microfluidic chip was developed that can double the total reagent volume and partitions compared to a single-layer microfluidic chip without changing the planar area. By employing black PDMS and a transparent glass sandwich structure, the fluorescence signal in different layers can be simply acquired without signal interference between the upper and lower chambers. Results confirm that the chip can realize uniform droplet segmentation, dPCR amplification, and fluorescence-signal detection. The double-deck chip can reach a 10^5^ dynamic range of detection for DNA in theory. In summary, the double-deck dPCR chip has several important features, including a simple operation process, highly uniform and stable digital partitions, and high throughput. It is our belief that this study not only resulted in the development of a dPCR chip, but also provides a route for other researchers to develop biochips. 

## Figures and Tables

**Table 1:** Comparation of double-deck dPCR chip and single-layer dPCR chip in sensitivity and dynamic range of detection.

	Low Limit of Detection (Copies/µL)	Uper Limit of Detection (Copies/µL)	Dynamic Range
Double-deck chip	0.091	1.35 × 10^4^	1.49 × 10^5^
Single-layer chip	0.181	1.26 × 10^4^	6.93 × 10^4^

## Supplementary Materials

The following are available online at https://www.mdpi.com/2072-666X/11/12/1025/s1, Figure S1: Schematic illustration of the fabrication process for the double-deck dPCR chip. (A) Preparing SU-8 master; (B) pouring PDMS over the master mold; (C) releasing the PDMS replica; (D) bonding the PDMS replica to a bottom glass slide by plasma treatment; (E) bonding the PDMS replica to a pre-punched top glass slide by plasma treatment; (F) bonding the PDMS-glass block and valves, Table S1: Sequences of primers and probes used in this experiment.
