# Supplementary material for: A Double-Deck Self-Digitization Microfluidic Chip for Digital PCR
Source: Micromachines (Basel). 2020 Nov 24;11(12):1025. doi: 10.3390/mi11121025 (PMC7759810; doi:10.3390/mi11121025)
Supplement: Supplementary file 1 [file micromachines-11-01025-s001.pdf]

Supporting Information

A double-deck self-digitization microfluidic chip for digital PCR

Gangwei Xu[a], Huangqing Si[a], Fengxiang Jing[b], Peng Sun[a], Zhao Dan[a], Dongping Wu[a]*

*[a]State Key Laboratory of ASIC and System, School of Microelectronics, Fudan University, Shanghai 200433,

[b]Shanghai Turtle Technology Company Limited, Shanghai 200439, China

*Corresponding author.

Email address: dongpingwu@fudan.edu.cn (D. Wu).

Chip mold fabrication

Standard multilayer soft lithography techniques were used for fabricating the double-deck self-digitization microfluidic chip. Two separate photoresist masks for microchannels and microchambers were designed using computer-aided design software Auto CAD (https://www.autodesk.com.cn/) and printed on transparency films. A 4-inch silicon wafer was washed ordinal with acetone, alcohol and deionized water by ultrasonic cleaning, and then baked at 200 °C for 10 min. Then a 30 μm thick layer of an SU-8 3050 negative photoresist (MicroChem Co., Ltd, USA) was spun on the cleaned silicon wafer at 3000 rpm for 30 s for making microchannels. The coated silicon wafer was exposed to ultraviolet radiation using a mask aligner (NXQ4006 Mask Aligner, California, USA). After exposure and development, the silicon wafer was hard baked on a hot plate at 200 °C for 30 min. Next, a 100 μm thick layer of an SU-8 3050 negative photoresist was used to construct the microchambers. After the features of the microchambers on the mask were aligned with the flow channels, the photoresist was exposed, baked and developed, until the mold

was finished. Lastly, the mold was hard baked at 200 °C for 1 hour.

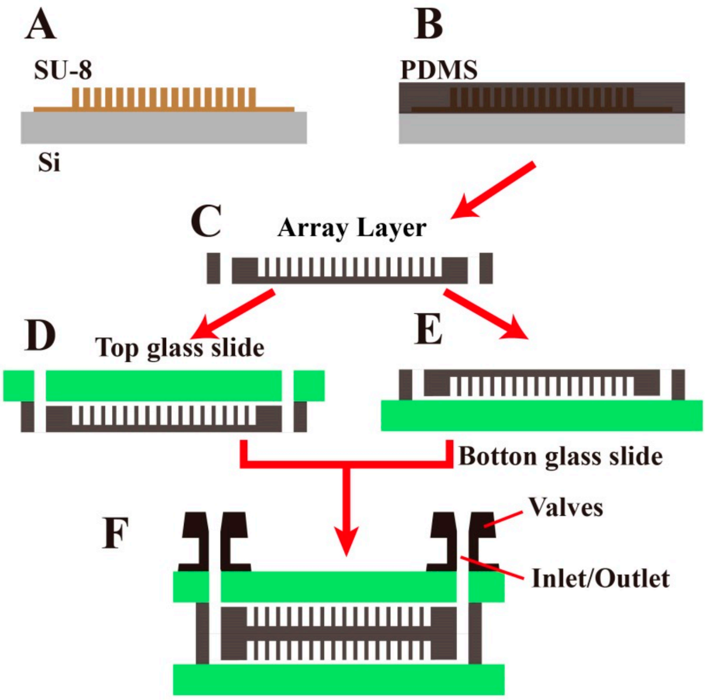

Fig. S1 Schematic illustration of the fabrication process for the double-deck dPCR chip. (a) Preparing SU-8 master; (b) pouring PDMS over the master mold; (c) releasing the PDMS replica (e) bonding the PDMS replica to a pre-punched top glass slide by plasma treatment; (d) bonding the PDMS replica to a bottom glass slide by plasma treatment (f) bonding the PDMS-glass block and valves.

Table S1. Sequences of primers and probes used in this experiment.

Forward primer	5'-CCTGCTGAAAATGACTGAA-3'
Reverse primer	5'-AAGATTTACCTCTATTGTTGG-3'
KRAS Reference probe	5'-CY5-AGAGTGCCTTGACGATACAGCTAA-AF594-3'

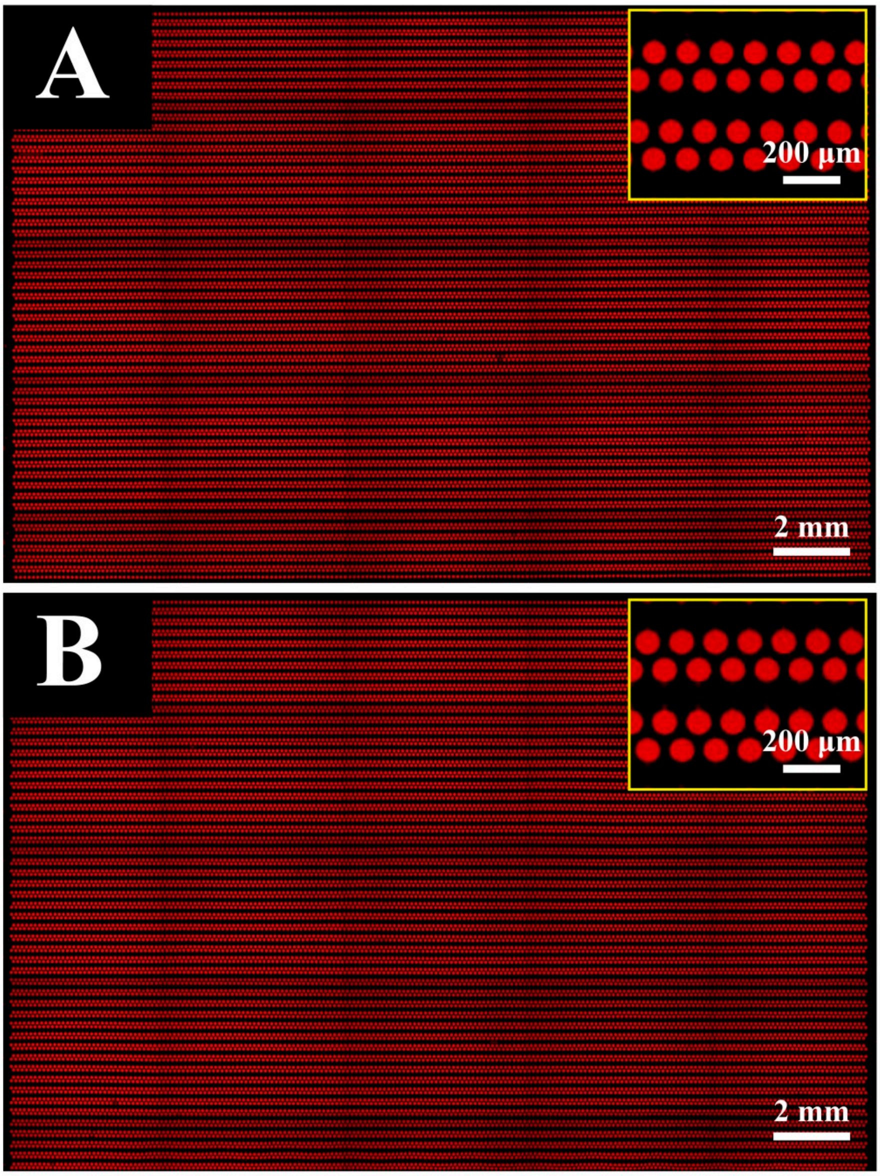

Fig. S2 Fluorescent image of the upper (A) and lower (B) arrays after loading fluorescence reagent.

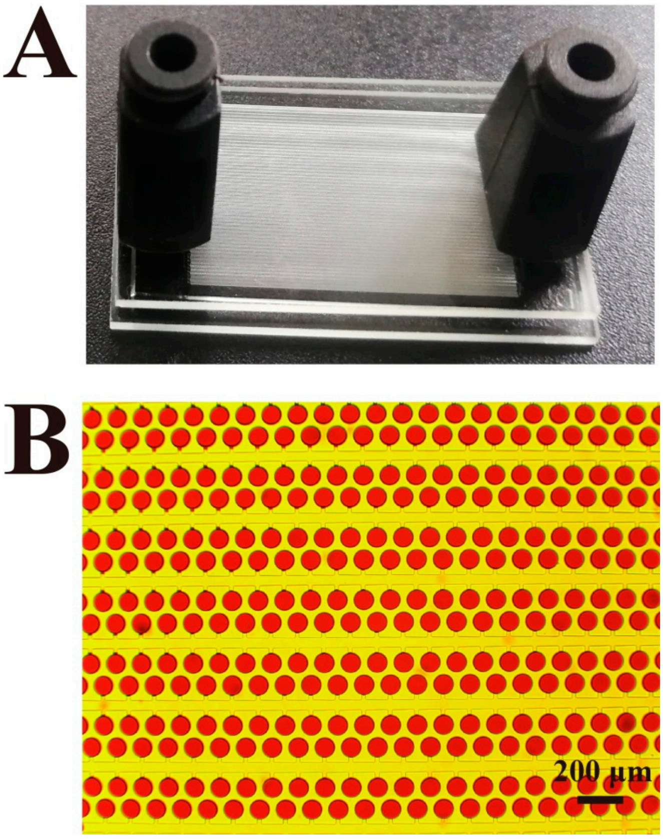

Fig. S3 (A) Photograph of the transparent PDMS chip and (B) micrograph of the chip after loading red dye reagent.
